# Age-Related Differences of Individuals’ Arithmetic Strategy Utilization with Different Level of Math Anxiety

**DOI:** 10.3389/fpsyg.2016.01612

**Published:** 2016-10-18

**Authors:** Jiwei Si, Hongxia Li, Yan Sun, Yanli Xu, Yu Sun

**Affiliations:** School of Psychology, Shandong Normal UniversityJinan, China

**Keywords:** math anxiety, strategy utilization, computational estimation, mental arithmetic, age-related differences

## Abstract

The present study used the choice/no-choice method to investigate the effect of math anxiety on the strategy used in computational estimation and mental arithmetic tasks and to examine age-related differences in this regard. Fifty-seven fourth graders, 56 sixth graders, and 60 adults were randomly selected to participate in the experiment. Results showed the following: (1) High-anxious individuals were more likely to use a rounding-down strategy in the computational estimation task under the best-choice condition. Additionally, sixth-grade students and adults performed faster than fourth-grade students on the strategy execution parameter. Math anxiety affected response times (RTs) and the accuracy with which strategies were executed. (2) The execution of the partial-decomposition strategy was superior to that of the full-decomposition strategy on the mental arithmetic task. Low-math-anxious persons provided more accurate answers than did high-math-anxious participants under the no-choice condition. This difference was significant for sixth graders. With regard to the strategy selection parameter, the RTs for strategy selection varied with age.

## Introduction

### Strategy Utilization and Arithmetic Performance

A strategy is “a procedure or a set of procedures for achieving a higher level goal or task” (Lemaire and Reder, 1999). The ability of individuals to effectively solve a problem depends primarily on the combination of information available for choosing and implementing the appropriate strategy. Siegler and Lemaire (1997) proposed a four-dimensional theoretical framework to explain how individuals utilize strategies, including strategy repertoire, strategy distribution, strategy execution, and strategy selection. Specifically, an examination of strategy execution focuses on efficiency (Imbo and LeFevre, 2009), and an investigation of strategy selection focuses on utilization. From a cognitive perspective, individual differences in arithmetic performance can be explained in terms of strategy utilization (Lemaire, 2010a). Lemaire (2010a) found that participants who were unable to use strategy efficiently were more likely to select a disadvantageous approach, which resulted in poor performance on an arithmetic task. Consistent with this result, Seaman et al. (2014) found that participants who select a simple, disadvantageous strategy during the first session performed poorer than others. Additionally, the ways in which arithmetic strategies are restricted by individual factors has become a focus of current research, particularly with regard to math anxiety (Imbo and Vandierendonck, 2007).

### Math Anxiety and Arithmetic Performance

Math anxiety, which is undue or excessive anxiety related to math, leads to physical, behavioral, and psychological changes that affect functioning in these domains. Such effects may appear in mathematics learning (Ashcraft and Krause, 2007), consumption decisions (Jones et al., 2012; Suri et al., 2013), and other areas. Young et al. (2012) found that math anxiety induced negative emotions. Evidence shows that math anxiety affects the ability to perform mental arithmetic (Ashcraft and Faust, 1994; Hopko et al., 2003) and computational estimation (Si et al., 2011). Si et al. (2011) found that math anxiety had a significant impact on computational estimation in two distinct contexts. The average reaction time (RT) of the low-math-anxiety group was significantly shorter than that of the middle- and high-math-anxiety groups. Additionally, the average accuracy of the high-math-anxiety group was the lowest among the three groups in both a pure digital and a word problem context. Wahid et al. (2014) revealed that math anxiety affected students’ performance, specifically, higher scores for math anxiety led to poorer performance in math course. Similarly, Andrews and Brown (2014) found that mathematics anxiety was negatively related to scores on standardized aptitude and achievement tests.

### Math Anxiety and Individual Arithmetic Strategies

From a cognitive perspective (Lemaire, 2010b), individual differences in math performance can be interpreted in terms of strategy utilization, and investigation of the characteristics of such utilization is among the advanced topics in this area (e.g., Chen et al., 2011). Studies have demonstrated that the utilization of arithmetic strategies depends on circumstances, individual characteristics, the questions involved, and so on, and math anxiety was one of the most important contributors to this phenomenon (Imbo and Vandierendonck, 2007).

Evidence has demonstrated that math anxiety affects the processes involved in mental estimation encoding, retrieval, and strategy selection (Cui et al., 2011). Mental estimation, one of the most widely discussed subjects, refers to the performance of arithmetic activities without the help of external instruments, and it includes the cognitive processes involved in encoding and other operations (Liu and Wang, 2008). Many studies have investigated this phenomenon from the perspective of the selection of mental estimation strategies (Núñez-Peña et al., 2006; Chen et al., 2011), and Imbo and Vandierendonck (2007) found that highly anxious individuals were less likely to choose strategies involving shortcuts to solve problems. Additionally, the effect of math anxiety depended on the difficulty of mental estimation problems (Seyler et al., 2003). Specifically, math anxiety had a minor effect on simple problems, but its effect increased as a function of the difficulty of problem (Wang and Liu, 2007). Moreover, the effects of math anxiety on strategy selection increased with age (Geng and Chen, 2005). Recent research has focused on the effects of problem characteristics, strategy characteristics, task circumstances, and participant characteristics on the selection of a computational estimation strategy (Hodzik and Lemaire, 2011; Si et al., 2012) as well as on identifying an efficient instrument with which to investigate the frequency of, diversity in, and variations in strategy utilization. Computational estimation, which involves the interaction of mental estimation, number conceptions, and arithmetic skills, refers to the process by which an individual uses her or his original knowledge to provide an imprecise answer to a problem (Si, 2002). Computational estimation is closely connected with mental estimation, as they involve common mental processes, although they are separate mental phenomena.

### Choice/No-Choice Method

The choice/no-choice method could obtain unbiased estimates of performance characteristics of strategies. As suggested by the name, the choice/no-choice method requires testing each participants under two types of conditions: conditions in which participants can freely choose which strategy to use (the choice condition) and conditions in which they must use a given strategy on all problems (the no-choice condition) (Siegler and Lemaire, 1997).

### Questions and Hypothesis

Several theories attempts to explain the effects of anxiety on arithmetic performance. According to attentional control theory, anxiety impairs the efficiency of two executive functions, including the inhibition and shifting functions. Additionally, high-anxious individuals often use compensatory strategies such as enhanced effort and use of processing resources to achieve a reasonable level of performance effectiveness (Eysenck and Derakshan, 2011). And the effects of math anxiety on age-related differences in the utilization of arithmetic strategies are worth discussing, and several preliminary explorations of the links between math anxiety and math strategies have already been conducted. Ashcraft and Faust (1994) found that high-anxiety individuals did not use self-terminating economic and time-saving strategies to finish verification tasks, reflecting their lack of flexibility and failure to adapt their strategy for use with complex mental arithmetic. Imbo and Vandierendonck (2007) found that fewer high-anxious children in the fourth, fifth, and sixth grades chose retrieval strategies compared with low-anxious children. Wu (2010) found that math anxiety affected the selection and execution of mental arithmetic strategies by third-grade children: low-anxiety children were more likely to select efficient retrieval strategies and worked rapidly and high accurately, whereas high-anxiety children were more likely to implement their strategy slowly and to have lower accuracy. Geng and Chen (2005) argued that the effects of math anxiety on arithmetic strategy selection vary among children and are more obvious among those in higher grades. Thus, the current study assumed that the effects of math anxiety on strategy utilization differed by age and that children would be more affected by this phenomenon than adults would.

Current research tends to use a mental arithmetic rather than a computational estimation task to explore the effects of math anxiety on strategy utilization (Wu, 2010; Chen et al., 2011). Computational estimation is considered one of the most effective tools for examining the flexibility and diversity that characterize individuals’ use of strategies (Si, 2002). Although computational estimation and metal arithmetic share several psychological processes, computational estimation is different from mental arithmetic. Specifically, mental arithmetic activates the left prefrontal cortex, whereas computational estimation primarily activates the bilateral parietal lobe (Dehaene et al., 1999; Lemer et al., 2003). Are there differences between effects of math anxiety on computational estimation and mental arithmetic? We currently lack adequate information to answer this question. Furthermore, the effects of math anxiety on the development of arithmetic strategies remain to be fully developed. Yet, it is important to examine the influence of math anxiety on individual development from childhood to adulthood. To this end, we must rely on both computational estimation and mental arithmetic, examining the execution and outcomes of different arithmetic calculation strategies according to the level of math anxiety of individuals to reveal changes in flexibility and in domain-specific and age-related strategy utilization. Thus, the present study assumed that both computational estimation and mental estimation were affected by math anxiety and that the strategy utilization of low-anxious individuals would be significantly superior to that of high-anxious individuals.

Evidence suggests that the way in which children utilize estimation strategies changes between fourth and sixth grades (Dowker, 1997; Lemaire et al., 2000). Ramirez et al. (2013) found a negative correlation between math anxiety and math scores in first and second graders, which showed that math anxiety had begun developing in children who had just entered school. Given that children in fourth grade have been learning two-digit addition, we included students in the fourth and sixth grades as well as adults as participants in our study to provide credible evidence of age-related differences in the effects of mathematics anxiety on certain problem-solving strategies. It is worth noting that math skills also affect mental arithmetic strategies (Imbo and Vandierendonck, 2007) and that the level of one’s computational estimation strategy increases as one’s numeracy skills develop (Dowker, 1997). To exclude the potential effects of differences in numeracy skills, this study used covariates to explore the specific factors influencing age-related differences in the effects of math anxiety on the utilization of arithmetic strategies.

## Materials and Methods

### Participants

A total of 203 undergraduates, 215 sixth-grade students, and 221 fourth-grade students from a city of Jinan in China completed group tests measuring math anxiety and math skills. All participants provided written informed consent. We divided these participants into high- and low-anxiety participants. Divide top and bottom 15% math scores into high and low anxiety participants, select 60 participants from each group students. After we eliminated invalid data, the final sample consisted of the following groups: adult high-anxiety group (*n* = 30: 14 male/16 female, *M* = 20.58 years), adult low-anxiety group (*n* = 30: 12 male/18 female, *M* = 20.57 years); sixth-grade high-anxiety group (*n* = 26: 11 male/15 female, *M* = 11.67 years), sixth-grade low-anxiety group (*n* = 30: 15 male/15 female, *M* = 11.64 years); fourth-grade group high-anxiety group (*n* = 27: 17 male/10 female, *M* = 9.63 years), fourth-grade low-anxiety group (*n* = 30: 14 male/16 female, *M* = 9.76 years). The average age of the adult group was 20.58 years, that of the sixth-grade group was 11.65 years, and that of the fourth-grade group was 9.65 years.

### Experimental Design

We used a 3 (age: fourth-grade students, sixth-grade students, adults) × 2 (math anxiety: high, low) × 2 (task type: computational estimation, mental arithmetic) × 3 (strategy utilization condition: choice, no-choice/1, no-choice/2) design. No-choice/1 and no-choice/2 indicated the no-choice/rounding-up and no-choice/rounding-down conditions, respectively, in the computational task, and they indicated the no-choice/partial-decomposition and no-choice/full-decomposition conditions, respectively, in the mental arithmetic task. Age and math anxiety were treated as between-subjects variables, and task type was treated as a within-subject variable. Data regarding RTs, accuracy, and strategy were recorded under each condition.

### Materials

#### Revised Mathematics Anxiety Rating Scale (R-MARS)

Participants completed the Revised Mathematics Anxiety Rating Scale (Liu, 2009, unpublished), a 21-item version of a widely used measure of math anxiety that asks respondents to indicate the degree to which different situations would make them anxious using a 5-point scale ranging from “not at all anxious” to “very anxious.” Higher scores reflected higher levels of math anxiety. The α coefficient of the original R-MARS was 0.932, and the α coefficient in this study was 0.94.

#### Math Anxiety Scale for Children

We used the 22-item amended version of the Math Anxiety Scale for Children (MASC) developed by Geng and Chen (2005). Our sample of children rated their level of anxiety in response to various activities on a 4-point scale on which 4 indicated “extremely nervous,” 3 indicated “very nervous,” 2 indicated “a little nervous,” and 1 indicated “not nervous.” The total score on the 22 items reflect a child’s level of mathematics anxiety, and higher scores reflect higher levels of math anxiety. The α coefficient of the scale ranged from 0.87 to 0.92. The α coefficient in this study was 0.903.

#### Arithmetic Skills Test

We used the French Kit (French et al., 1963) version of this standardized paper-and-pencil test, which includes one page of complex addition problems and one page of complex subtraction and multiplication problems. Each page contains six rows of 10 vertically oriented problems, and each participant was given 2 min per page to solve the problems as quickly and accurately as possible. Total arithmetic scores are calculated based on the number of correct answers, and higher scores reflect higher levels of arithmetic skills.

#### Arithmetic Calculations

The computational estimation and mental arithmetic tasks were the equivalent of 84 two-digit addition problems. The unit digit of one operand was larger than 5, that of the other operand was smaller than 5, and the sum consisted of three digits. Half of the problems did not involve carrying a number from the units to the 10s (e.g., 34 + 21), and the other half did involve such a carry (e.g., 16 + 38). A total of 84 problems were divided into three blocks, and each strategy-utilization condition included 28 problems. Problems were selected to control for variables that crucially influence arithmetic performance (Geary, 1996 for reviews). We ensured that (a) no operand included the digit 0 or 5 (e.g., 20 or 35), (b) no problems included the same tens digit (e.g., 73 + 76), (c) no operands included a repeated digit (e.g., 44 + 79), (d) no problems included reversed operands (e.g., if 73 + 58 were used, 58 + 73 was not), (e) half of the larger operands were presented on the right and the other half were presented on the left, and (f) half of the larger units of operands were presented on the right side and the other half were presented on the left.

### Experimental Procedures

**FIGURE 1:**
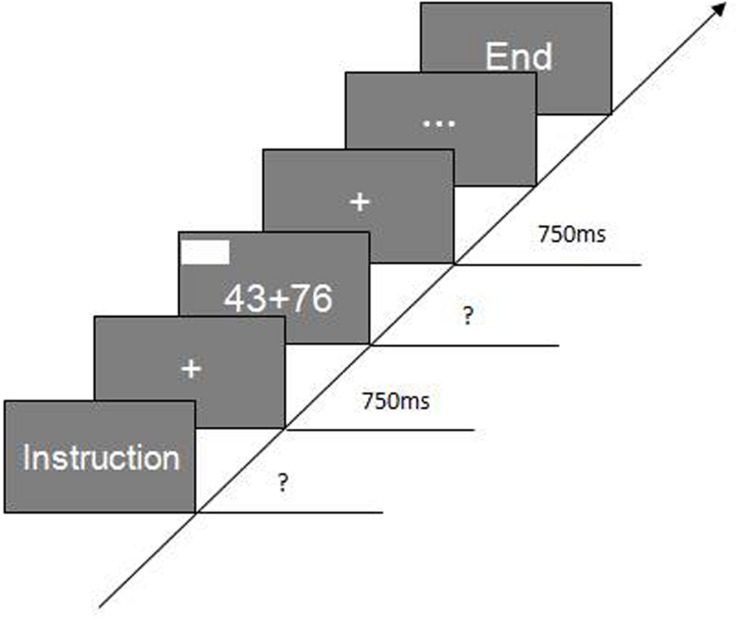
**Experimental procedures**.

The experiment was conducted in a quiet room. The computational estimation test was administered first, followed by the mental arithmetic test. Each test lasted approximately 45–60 min; the two tests were conducted in the morning and the afternoon of the same day or during 2 days. Two computational estimation strategies were presented to the participants before the computational estimation test: rounding up and rounding down. The rounding-down strategy was described as rounding both operands down to the nearest smaller decade (e.g., 30 + 50 to estimate 32 + 56). The rounding-up strategy was described as rounding both operands up to the nearest larger decade (e.g., 40 + 60 to estimate 32 + 56). The participants were then informed that three strategy conditions would be used in this test: the choice condition (C1), in which one strategy must be chosen for each question to estimate the correct answer as closely as possible; the no-choice/rounding-up condition (C2), in which all questions must be answered using the rounding-up strategy; and the no-choice/rounding-down condition (C3), in which all questions must be answered by using the rounding-down strategy. Stimuli were presented in 42-point Times New Roman font at the center of a 13-inch computer screen controlled by a Lenovo B450 laptop. The experiment was controlled by E-Prime software. The program generated the displays and recorded latencies to the nearest millisecond. The experimental procedures were the same under each experimental condition: (a) the number of the participants was entered into the computer; (b) instructions were presented at the middle of the screen; (c) participants began after they understood the instructions; (d) each trial started with a fixation point “+,” which was displayed for 750 ms; (e) after the fixation point disappeared, the question appeared, the time to answer each question was recorded, and participants pressed the “Enter” key to stop the timing; and (f) the next trial began. After a practice exercise, participants began the formal experiment, which followed the same procedures. The test order was C1→C2→C3, and participants rested for 5 m at the end of each experimental condition (**Figure 1**).

Before the beginning of the mental arithmetic test, two strategies were presented: the full-decomposition strategy, which involved splitting off the 10s and the units in both integers and adding (e.g., 73 + 58 = _; 70 + 50 = 120, 3 + 8 = 11, 120 + 11 = 131), and the partial-decomposition strategy, which involved adding first the 10s and then the units of the second integer to the first un-split integer (e.g., 73 + 58 = _; 73 + 50 = 123, 123 + 8 = 131). The participants were informed that three conditions would be used in this test: the choice condition (C1), in which respondents chose which of the two strategies was the quickest way to solve the problem; the no-choice/partial-decomposition strategy (C2), in which all the questions had to be solved used the partial-decomposition strategy; and the no-choice/full-decomposition strategy (C3), in which all the questions had to be solved using the full-decomposition strategy. The test procedures were almost the same as those used in the computational estimation test; only C1 differed slightly: after participants input their answers and pressed Enter, one question appeared: “Which strategy did you use to solve the problem? (1) The partial-decomposition strategy or (2) The full-decomposition strategy.” Participants were asked to respond truthfully and continue to the next question. The order and rest time were identical those used during the computational estimation test.

### Data Processing

The experimental data were analyzed with repeated-measures ANOVAs with SPSS 17.0. There were no missing data in this study. The reasons are as follows: first of all, in order to ensure participants can complete all trials, stimulates would not disappear until participants press the key. Secondly, we arranged experiments base on participants’ available time, so all participants selected took part in this study and never gave up during the process of study.

## Results

### Strategy Execution

Strategy execution refers to the speed and accuracy with which individuals solve problems when they must use specific strategies to do so. The results of our experiment are presented in **Tables 1** and **2**.

**Table 1:** Response time (RT) and accuracy of participants in computational estimation strategy use *M*(*SD*).

Grade group	Math anxiety level	Mean RT (ms)			Accuracy (%)		
		C1	C2	C3	C1	C2	C3
Grade four group	High anxiety	10789 (3441)	9608 (2745)	5650 (2069)	61.41 (12.63)	92.10 (6.69)	96.85 (3.33)
	Low anxiety	11599 (3978)	8107 (2400)	5060 (1753)	61.97 (16.23)	94.39 (5.44)	98.45 (2.60)
Grade six group	High anxiety	9688 (4594)	7096 (2411)	4489 (1709)	61.24 (16.45)	94.17 (6.61)	96.88 (3.82)
	Low anxiety	8870 (3637)	5816 (2222)	3437 (1313)	75.75 (15.36)	97.25 (3.60)	98.81 (2.17)
Adult group	High anxiety	6071 (1131)	3813 (768)	2583 (557)	83.93 (10.84)	96.43 (4.19)	98.22 (1.9)
	Low anxiety	6100 (1586)	3935 (754)	2334 (335)	85.03 (12.21)	96.60 (3.03)	98.30 (2.91)

**Table 2:** Response time (RT) and accuracy of participants in mental arithmetic strategy use *M*(*SD*).

Grade group	Math anxiety level	Mean RT (ms)			Accuracy (%)		
		C1	C2	C3	C1	C2	C3
Grade four group	High anxiety	12458 (4300)	11546 (3409)	9343 (2029)	92.49 (7.09)	91.74 (5.56)	93.09 (7.34)
	Low anxiety	11009 (3295)	10928 (3733)	9316 (2875)	92.10 (7.96)	91.46 (9.40)	91.25 (10.34)
Grade six group	High anxiety	9878 (3470)	9183 (2883)	7645 (1974)	88.89 (9.51)	88.90 (10.75)	89.01 (9.45)
	Low anxiety	7228 (2643)	7290 (3410)	6091 (2149)	91.17 (7.49)	93.48 (4.72)	94.62 (5.53)
Adult group	High anxiety	5324 (1080)	5289 (1240)	4334 (855)	94.80 (4.03)	94.74 (4.29)	96.22 (3.21)
	Low anxiety	5395 (820)	5171 (1207)	4351 (710)	95.74 (4.46)	94.56 (4.65)	97.26 (2.96)

We analyzed the response times (RTs) and accuracy of two computational estimation no-choice conditions with repeated-measures ANOVAs using a 3 (age group: fourth and sixth graders, adults) × 2 (math anxiety: high and low anxiety) × 2 (no-choice conditions) design with arithmetic skill as the covariate (**Table 3**). The results were as follows: (a) RTs: The main effect under the no-choice condition was significant [*F*_(1,166)_ = 58.81, η^2^ = 0.262, *p* < 0.001], and the rounding-up strategy required more time than the rounding-down strategy (6324 and 3883 ms, respectively). The main effect of age group was significant, *F*_(2,166)_ = 12.78, η^2^ = 0.133, *p* < 0.001. Fisher’s Least Significant Difference (LSD) test was applied in *post hoc* analyses due to its higher sensitive and convenience. *Post hoc* analyses revealed no significant difference between adults and sixth-grade students, whereas significant differences between the other pairs of groups were observed [*p*_(adults, sixth graders)_ = 0.720, *p*_(adults, fourth graders)_ = 0.007, *p*_(sixth graders, fourth graders)_ < 0.001]. We also used Bonferroni method to check our findings. Bonferroni method found the same results. There is no significant difference between adults and sixth-graders [*p*_(adult, sixth graders)_ = 1 > 0.05], whereas significant difference between the other pairs of groups were found [*p*_(adult, fourth graders)_ = 0.02 < 0.05, *p*_(sixth graders, fourth graders)_ < 0.001]. The main effect of math anxiety was significant, *F*_(1,166)_ = 6.11, η^2^ = 0.036, *p* = 0.014, as the high-anxiety group was slower than the low-anxiety group (C2: 6727 and 5952 ms, respectively; C3: 4178 and 3610 ms, respectively). No interaction was found for the following. (b) Accuracy: the main effect of math anxiety was significant, *F*_(1,166)_ = 8.45, η^2^ = 0.048, *p* = 0.004; the low-anxiety group was more accurate that the high-anxiety group (C2: 94.31 and 96.08%, respectively; C3: 97.35 and 98.52%, respectively). The interaction between the non-choice condition and age group was significant, *F*_(2,166)_ = 3.42, η^2^ = 0.040, *p* = 0.035. Simple-effect analysis revealed no significant difference between adults and sixth graders, whereas significant differences between the other pairs of groups were observed [*p*_(adults, sixth graders)_ = 0.468, *p*_(adults, fourth graders)_ = 0.001, *p*_(sixth graders, fourth graders)_ = 0.010]. The effect of age group was not significant under C3, *F*_(2,166)_ = 0.56, *p* = 0.572.

**Table 3:** Repeated-measures ANOVAs of computational estimation strategy execution.

	Mean RT (ms)		Accuracy (%)	
	*F*	*p*	*F*	*p*
The no-choice condition	58.81	0.000^∗∗∗^	1.56	0.214
Grade group	12.78	0.000^∗∗∗^	1.88	0.156
Math anxiety	6.11	0.014^∗^	8.45	0.004^∗∗^
Grade group × the no-choice condition	2.80	0.084	3.42	0.035^∗^
Math anxiety × the no-choice condition	0.30	0.586	0.83	0.363
Math anxiety × Grade group	0.18	0.833	1.66	0.194
Math anxiety × Grade group × the no-choice condition	1.55	0.216	0.22	0.802

*^∗^p < 0.05; ^∗∗^p < 0.01; ^∗∗∗^p < 0.001.*

**FIGURE 2:**
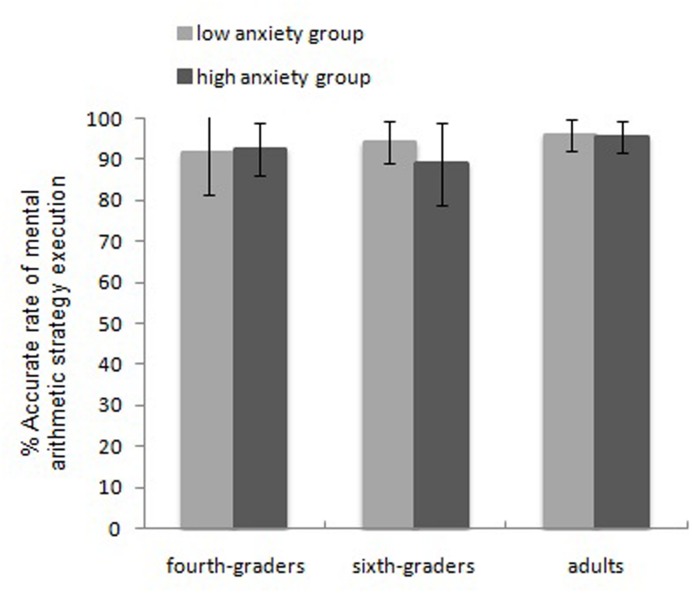
**Interaction of grader groups and math anxiety in mental arithmetic strategy execution**.

The RTs and accuracy of the two mental arithmetic no-choice conditions were analyzed separately with repeated-measures ANOVAs with a 3 (age group: fourth and sixth graders, adults) × 2 (math anxiety: high and low anxiety) × 2 (no-choice condition) design treating arithmetic skill as the covariate (**Table 4**). This analyses revealed the following: (a) RTs: the main effect of the no-choice condition was significant, *F*_(1,166)_ = 27.45, η^2^ = 0.142, *p* < 0.001, and the partial-decomposition strategy required more time than the full-decomposition strategy (8155 and 6785 ms, respectively). The main effect of age group was significant, *F*_(2,166)_ = 13.22, η^2^ = 0.137, *p* < 0.001, as adults were faster than the sixth graders, and sixth graders were faster than fourth graders (C2: 5230, 8169, and 11221 ms, respectively; C3: 4342, 6812, and 9329 ms, respectively). Fisher’s LSD test analyses revealed no significant difference between adults and sixth graders, whereas significant differences between other pairs of groups were observed [*p*_(adults, sixth graders)_ = 0.247, *p*_(adults, fourth graders)_ = 0.041, *p*_(sixth graders, fourth graders)_ < 0.001]. Bonferroni test revealed no significant difference between adults and sixth graders [*p*_(adults, sixth graders)_ = 0.74 > 0.05], whereas significant differences between fourth graders and sixth graders [*p*_(fourth graders, sixth graders)_ < 0.001], These results are consistent with LSD test findings. Additionally, Bonferroni test found no significant difference between adults and fourth graders [*p*_(adults, fourth graders)_ = 0.12 > 0.05], this is not consistent with LSD test. The main effect of math anxiety was not significant, *F*_(1,166)_ = 1.23, η^2^ = 0.007, *p* = 0.270. No interaction was found. (b) Accuracy: only the interaction between math anxiety and age group was significant, *F*_(2,166)_ = 2.93, η^2^ = 0.034, *p* = 0.056. According to **Figure 2**, the simple-effect analysis revealed no significant difference between the high- and low-anxiety groups among adults, *F* < 1 (C2: 94.74 and 94.56%, respectively; C3: 96.22 and 97.26%, respectively); the accuracy of the high-anxiety group was lower than that of the low-anxiety group among sixth graders, *F*_(1,54)_ = 6.87, *p* = 0.011 (C2: 88.90 and 93.48%, respectively; C3: 89.01 and 94.62%, respectively); no significant difference was found between the high- and low-anxiety groups among fourth graders, *F* = 0.26 (C2: 91.74 and 91.46%, respectively; C3: 93.09 and 91.25%, respectively).

**Table 4:** Repeated-measures ANOVAs of mental arithmetic strategy execution.

	Mean RT (ms)		Accuracy(%)	
	*F*	*p*	*F*	*p*
The no-choice condition	27.45	0.000^∗∗∗^	0.03	0.869
Grade group	13.22	0.000^∗∗∗^	0.60	0.553
Math anxiety	1.23	0.270	1.38	0.242
Grade group × the no-choice condition	1.28	0.281	0.18	0.836
Math anxiety × the no-choice condition	0.48	0.489	0.04	0.842
Math anxiety × Grade group	1.02	0.364	2.93	0.056^∗^
Math anxiety × Grade group × the no-choice condition	0.23	0.799	1.04	0.355

*^∗^p < 0.05; ^∗∗^p < 0.01; ^∗∗∗^p < 0.001.*

### Strategy Selection

**FIGURE 3:**
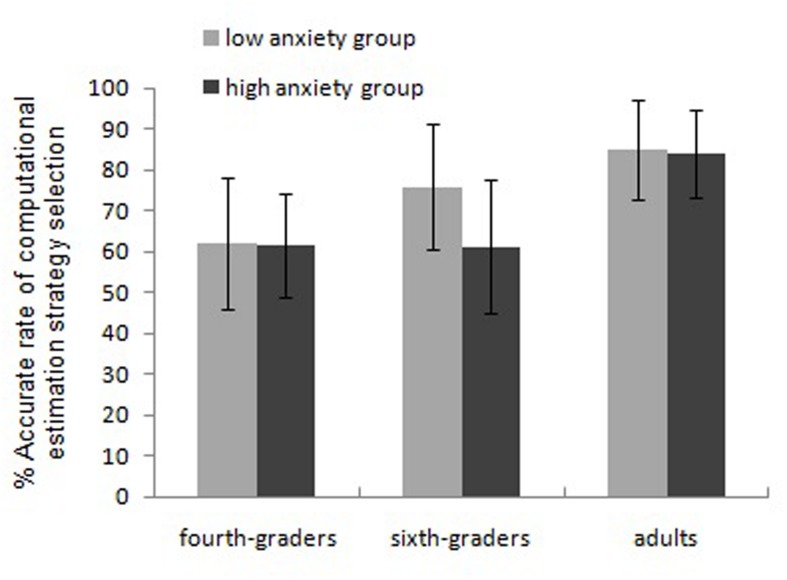
**Interaction between grade groups and math anxiety in computational estimation strategy selection**.

The results of the computational estimation test under the choice condition reflected the choice of a strategy. If the result of the chosen strategy was close to the correct result, we regarded the strategy as correct; the accuracy of a strategy choice was the rate at which a correct strategy was selected. The RTs and accuracy rates of the three groups of participants are presented in **Table 1**. We analyzed RTs and accuracy separately using repeated-measures ANOVAs with a 3 (age group: fourth and sixth graders, adults) × 2 (math anxiety: high and low anxiety) design, treating arithmetic skill as the covariate. This analysis revealed the following: (a) RTs: the main effect of age group was marginally significant, *F*_(2,166)_ = 2.57, η^2^ = 0.030, *p* = 0.79. An LSD test analyses showed that adults (6086 ms) were much faster than sixth graders (9250 ms), and sixth graders were much faster than fourth-grade students (11215 ms). These results are consistent with Bonferroni test findings. The main effect of math anxiety was not significant, *F*_(1,166)_ = 0.36, η^2^ = 0.002, *p* = 0.55, and the interaction between age group and math anxiety was not significant, *F*_(2,166)_ = 0.99, η^2^ = 0.012, *p* = 0.374. (b) Accuracy: the main effect of age group was significant, *F*_(2,166)_ = 14.67, η^2^ = 0.150, *p* < 0.001, as the accuracy rate of adults (84.48%) was much higher than that of sixth-grade students (69.02%), and that of the latter group was much higher than that of fourth-grade students (61.70%). The main effect of math anxiety was significant, *F*_(1,166)_ = 6.59, η^2^ = 0.038, *p* = 0.011, as the accuracy rate of the low-anxiety group was lower than that of the high-anxiety group (69.50 and 74.25%, respectively). The interaction between age group and math anxiety was significant, *F*_(2,166)_ = 4.60, η^2^ = 0.053, *p* = 0.011. **Table 2** presents the simple-effect analysis, which suggests the following: (a) Among adults, no significant difference was observed in the accuracy of the strategy choices of the low- and high-anxiety groups, with both groups being highly accurate (high anxiety: 83.93%, low anxiety: 85.03%); (b) Among sixth-grade students, the accuracy of the high-anxiety group’s strategy choice was much lower than that of the low-anxiety group (61.24 and 75.75%, respectively); (c) Among fourth graders, the accuracy rate of the strategy choice was low for both groups (61.41 and 61.97% for high- and low-anxiety groups, respectively), and the groups did not differ significantly (*t*_adults_ = -0.37, *p* = 0.713; *t*_sixth graders_ = -3.41, *p* = 0.001; *t*_fourth graders_ = -0.14, *p* = 0.886) (**Figure 3**).

The accuracy score on the mental arithmetic task was calculated as the percentage of correct answers produced by the use of one of the mental arithmetic strategies. The RTs and accuracy rates are shown in **Table 2**. We analyzed the RTs and accuracy rates for each choice condition separately using repeated-measures ANOVAs with a 3 (age group: fourth and sixth graders, adults) × 2 (math anxiety: high and low anxiety) design, treating arithmetic skill as the covariate. The results were as follows: (a) RTs: the main effect of age group was significant *F*_(2,166)_ = 10.4, η^2^ = 0.111, *p* < 0.001. An LSD test analyses showed no significant difference between adults and sixth graders [*p*_(adults, sixth graders)_ = 0.84], whereas significant difference between adults and fourth graders [*p*_(adults, fourth graders)_ < 0.05], sixth graders and fourth graders [*p*_(sixth graders, fourth graders)_ < 0.001]. Specifically, sixth graders (7663 ms) were much faster than adults (7810 ms), who were much faster than fourth graders (9977 ms). Bonferroni test revealed the same findings [*p*_(adults, sixth graders)_ = 1 > 0.05, *p*_(adults, fourth graders)_ = 0.034 < 0.05, *p*_(sixth graders, fourth graders)_ < 0.001, respectively]. The main effect of math anxiety was significant, *F*_(1,166)_ = 5.84, η^2^ = 0.034, *p* = 0.017, as the high-anxiety group spent more time choosing a strategy (high-anxiety group: 9071 ms, low-anxiety group: 7877 ms). The interaction between age group and math anxiety was not significant, *F*_(2,166)_ = 0.73, η^2^ = 0.009, *p* = 0.483. (b) Accuracy: only the main effect of age group was marginally significant, *F*_(2,166)_ = 2.97, η^2^ = 0.035, *p* = 0.054. Fisher’s LSD test analyses revealed no significant difference between adults and fourth graders, adults and sixth graders, whereas significant differences were found between fourth graders and sixth graders [*p*_(adults, fourth graders)_ > 0.05, *p*_(adults, sixth graders)_ > 0.05, *p*_(sixth_ _graders, fourth_ _graders)_ < 0.05; 92.8, 90.9, and 94.1% for adults, sixth graders, and fourth graders, respectively]. Bonferroni test found no significant among ages [*p*_(adults, fourth graders)_ = 1 > 0.05, *p*_(adults, sixth graders)_ = 1 > 0.05, *p*_(sixth graders, fourth graders)_ = 0.06 > 0.05]. The main effect of math anxiety was not significant, *F*_(1,166)_ = 0.28, η^2^ = 0.002, *p* = 0.596, and the interaction between math anxiety and age group was also not significant, *F*_(2,166)_ = 0.53, η^2^ = 0.006, *p* = 0.590.

### Adaptiveness of Strategy Choice

Following prior research (Imbo and LeFevre, 2011), we defined the adaptiveness of a computational estimation strategy choice as follows: if the estimate produced by the chosen strategy was close to the correct answer, the choice of participants was adaptive. According to this definition, under C1, the accuracy rate of strategy use was the index of the adaptiveness of the strategy choice. In the context of the foregoing analysis, we can conclude the following: (1) adults’ strategy choices were more adaptive than were those of children, and adults’ choices were not influenced by math anxiety; (2) fourth-grade students’ strategy choices were less adaptive than adults, but these choices were not influenced by math anxiety; and (3) the adaptiveness of sixth graders’ strategy choices was influenced by math anxiety, with the low-anxiety group making more adaptive choices.

Following previous research (e.g., Imbo and LeFevre, 2009), we determined the best mental arithmetic strategy based on the performance of participants under the no-choice condition. In this context, the strategy that can be implemented most quickly is the best strategy. The percentage of participants utilizing the best strategy was the index of the adaptiveness of the mental arithmetic strategy under the choice condition. Analysis of variance using a 3 (age group) × 2 (math anxiety) design to examine the percentage of those using the optimal strategy revealed that neither the main effect nor the interaction was significant.

## Discussion and Conclusion

### Why Math Anxiety Affects the Use of Arithmetic Strategies

The present study found that math anxiety affected the choice of the strategy used for computational estimation and mental arithmetic processing. These results can be explained from a variety of perspectives. (1) According to cognitive interference theory (Northern, 2010), people with high levels of anxiety are concerned about others’ evaluation of them while they are performing tasks. This generates evaluation anxiety, which leads to negative self-statements, which diverts working memory resources from the central executive system and the phonological loop, resulting in poor performance. (2) According to processing efficiency theory (Eysenck et al., 2007), working memory resources are limited. When people are anxious, their anxiety occupies part of their working memory resources, thereby reducing the resources available to process the current tasks, leading to a reduction in the efficiency of cognitive processing. According to this view, math anxiety occupies working memory resources and thereby affects individuals’ cognitive performance (Cui et al., 2011). Si et al. (2014) provided evidence that highly anxious individuals had a higher working memory load than did individuals with low levels of anxiety. In terms of strategy-switch costs, participants must inhibit the strategy they just executed and activate a new strategy when selecting a strategy for a new problem (Lemaire, 2010b), and this process occupies additional working memory resources. Anxiety, strategy-switching, and cognitive tasks compete for limited cognitive resources, resulting in poor performance in people with high levels of anxiety. (3) According to attentional control theory (Eysenck and Derakshan, 2011), people with high levels of math anxiety transfer their focus from the arithmetic tasks (i.e., the goal-directed attentional system) to mathematics anxiety (i.e., the stimulus-directed attentional system), resulting in their poor performance on arithmetic tasks. According to many studies (e.g., Derakshan and Eysenck, 2009), this imbalance leads directly to negative effects on inhibition and the shifting function. According to this theory, the suppression and conversion functions of the central executive are more susceptible to math anxiety compared with updating and dual-task coordination functions. Issues regarding strategy, especially strategy selection, are closely related to suppression and conversion functions; therefore, the strategy utilization of people with high levels of anxiety is inferior. (4) According to inhibition theory (Hopko et al., 1998), people with high levels of anxiety have difficulty inhibiting intrusive anxious thoughts when performing arithmetic tasks, which hinders their performance on those tasks. Si et al. (2014) also found that participants who were highly anxious about math selected their strategy more slowly because of the effects of anxiety. Based on the foregoing, we can conclude that different components of the working memory system are affected differently by anxiety and that the phonological loop and central executive components (the suppression and conversion functions) are especially susceptible to anxiety. We speculate that mathematical problem-solving situations generate math anxiety, which interferes with the working memory system required to solve such problems. Math anxiety affects the working memory system (especially the phonological loop and the central executive component), thereby influencing the process by which a strategy is implemented, which ultimately impacts performance.

### The Specific Impact of Math Anxiety on the Use of Computational Estimation and Mental Arithmetic Strategies

This study found that math anxiety affects the use of computational estimation and mental arithmetic strategies in different ways. Specifically, math anxiety affects the execution of strategies for computational estimation but not those for mental arithmetic. In terms of strategy choice, math anxiety affects the adaptiveness of the choice of computational estimation strategies and the speed with which a mental arithmetic strategy is chosen. This shows that the speed–accuracy trade-off related to strategy selection differs for the two tasks: individuals finish computational estimation tasks quickly at the expense of accuracy, whereas they perform mental calculation accurately at the expense of speed, indicating that the effect on strategy choice is domain specific. There are several reasons for these differences. First, computational estimation and mental arithmetic constitute two different forms of arithmetic based on significantly different physiological substrates. Second, individuals are usually not familiar with computational estimation, as they tend to focus on written and oral calculation in daily life. Third, the difficulty of the task may also be relevant. The performance of computational estimation tasks requires participants to use only rounding-down and rounding-up strategies, but the problems are mixed (i.e., the unit of one operand is larger than 5, and the unit of the other is smaller than 5); thus, the results produced by a mixed strategy were closer to the correct answers. Participants (especially those who do not know the rules) find it difficult to make choices while solving a variety of different problems, as these choices require more cognitive resources. From this perspective, the computational estimation tasks used in this study were more difficult the than mental calculation ones.

### Age Differences in the Effects of Math Anxiety on the Use of Arithmetic Strategies

The results show that the impact of math anxiety on math strategies significantly differs by age. This difference is reflected in the strategy selection for computational estimation tasks and in the accuracy of mental arithmetic tasks. It is more difficult to choose strategies for computational estimation than for mental arithmetic tasks; in terms of strategy execution, mental arithmetic tasks are more difficult than computational estimation tasks because mental arithmetic tasks involve two-digit addition, whereas computational estimation involves only one-digit addition. This phenomenon can be interpreted in terms of processing efficiency theory. Attentional resources are limited, and task performance is worse when arithmetic tasks are difficult (Eysenck et al., 2007). Depending on the results, in the strategy selection for computational estimation tasks and in the accuracy of mental arithmetic tasks, only sixth-grade children were constrained by math anxiety, and students with higher levels of anxiety devoted more attention to speed and sacrificed accuracy. It is possible that fourth-grade students were at the primary stage of math anxiety (Ashcraft and Moore, 2009) and were therefore less affected by such anxiety. As individuals age, they accumulate knowledge, and the difficulty of the material they learn increases; thus, their experience with and the effect of math anxiety increases, leading to poor performance by those with high levels of anxiety. These results are consistent with previous findings: high- and low-anxiety adults differed in their performance of complex but not of simple arithmetic tasks (Imbo and Vandierendonck, 2007). The adults in the present study were less affected by math anxiety, which can be interpreted in terms of processing efficiency theory. Due to the maturity of their cognitive development, adults found the arithmetic tasks in the study easier and had to use fewer cognitive resources than was the case with the children. Thus, even adults with high levels of anxiety had sufficient resources to solve the problems. This observation is consistent with previous findings showing that people with high math anxiety perform worse on complex arithmetic tasks, but that they perform at the same level as people with lower levels of math anxiety on simple tasks (Imbo and Vandierendonck, 2007). Siegler and Lortie-Forgues (2014) suggested that individual skills at magnitude representation gradually improve with age and that experiences that foster magnitude representation also improve other numerical skills, such as arithmetic learning. However, Imbo and Vandierendonck (2007) found that math anxiety had significant effects on the selection and utilization of simple arithmetic strategies. These discrepant results may be attributable to cultural differences between the East and West, as evidence shows that Chinese participants had better computational skills than did Belgian and Canadian participants (Imbo and LeFevre, 2009, 2011). Cultural differences in the behaviors involved in computational estimation strategies were also investigated except mental arithmetic strategy behaviors (Imbo and LeFevre, 2011). Consistent with this view, Xu et al. (2014) found that Chinese participants were more efficient than Belgian and Canadian participants, but their choices were less adaptive. One possible explanation for these cultural differences is that Chinese, Belgian, and Canadian students have different educational experiences. Indeed, Chinese individuals may be less tolerant than those from other cultures of approximate solutions. Hence, when asked to perform rounding strategies, Chinese participants may have to inhibit their tendency to perform exact calculations, a process that consumes working memory resources. In contrast, educational reform movements in European countries over the last 20 years have emphasized flexibility, adaptive expertise, and the use of metastrategies as part of children’s learning about arithmetic (Verschaffel et al., 2009). Thus, Belgian and Canadian students are likely to be quite familiar with using a variety of strategies and capitalizing on the most appropriate one.

### Limitations and Future Research

The present research has several limitations. First, the range of participants is not widely enough. Fourth and sixth graders are relatively close in age and perhaps in learning progression. Wigfield and Meece (1988) found ninth-grade students reported experiencing the most worry about math and sixth graders the least. So future researches should pay attention to younger math learners or middle school or high school participants. That might speak more to the developmental progression. Second, we didn’t use oral report. So although we asked participants to use rounding strategy we can’t ensure the process of participants computational estimation or mental arithmetic. Third, it may be a problem to analyze the adult data with the Grade 4 and 6 data given that the adult had close to ceiling performance. Moreover, Krinzinger et al. (2009) revealed a close relationship between math anxiety and math ability on evaluation of mathematics in primary school children and math anxiety did not exert direct effects on math ability. So further researches should pay more attention to mediators between math anxiety and math performance.

Despite these limitations, this study addresses some key issues in the current literature on math anxiety. First of all, the present study revealed that the effect of math anxiety on computational estimation was more pronounced than that on mental arithmetic. This may be due to the fact that students received more formal training in mental arithmetic at school. Imbo and Vandierendonck (2008) found practice effects on strategy selection and strategy efficiency for simple mental arithmetic problems. Thus appropriate practice and educational intervention may promote the development of children’s computational estimation strategy choice. Secondly, sixth-grade students with lower arithmetic skills are more strongly affected by math anxiety than adults with higher arithmetic skills. In other words, improving arithmetic skills can reduce math anxiety. This also suggests that education, learning, and practice play key roles in strategy development. Finally, this study found that the effect of math anxiety on arithmetic strategy utilization may change with age. It is consistent with findings in Lemaire’s study (Lemaire, 2010a). Specifically, students in lower grade were less affected by math anxiety, but this impact gradually increased as students progressed through school. Subsequently, the impact of math anxiety would decrease slightly with the development of cognitive function and more skilled at arithmetic (Lemaire, 2010a). So we can suppose that the effect of math anxiety on the utilization of relevant strategies may follow an unstable inverted U-shaped trend over the course of individual development due to external factors (e.g., the math curriculum), internal factors (e.g., cognitive development) (Ramirez et al., 2016) and their interactions. But there is a need for systematic studies to investigate the trend due to the limitation of our samples.

Issues related to students’ mathematics performance are widely discussed. Because mathematics is a compulsory subject in higher-level institutions, especially in courses of study in science and technology, failure in that subject may result in delayed graduation or dismissal from a university. Hence, future researches should focus on the development of students’ strategies to improve their flexibility via practice.

## Ethical Standards

All procedures performed in studies involving human participants were in accordance with the ethical standards of the ethics committee on human experimentation of Shandong Normal University and with the 1964 Helsinki declaration and its later amendments or comparable ethical standards.

## Author Contributions

JS and HL made substantial contributions to the conception, design of the work, the acquisition, analysis, interpretation of data for the work; and drafting the work, revising it critically for important intellectual content; and final approval of the version to be published; and agreement to be accountable for all aspects of the work in ensuring that questions related to the accuracy or integrity of any part of the work are appropriately investigated and resolved. YaS made substantial contributions to the conception or design of the work, or the acquisition, analysis, or interpretation of data for the work; and revising it critically for important intellectual content; and Final approval of the version to be published; and Agreement to be accountable for all aspects of the work in ensuring that questions related to the accuracy or integrity of any part of the work are appropriately investigated and resolved. YX and YuS made substantial contributions to the acquisition, analysis, or interpretation of data for the work; and revising it critically for important intellectual content; and final approval of the version to be published; and agreement to be accountable for all aspects of the work in ensuring that questions related to the accuracy or integrity of any part of the work are appropriately investigated and resolved.

## Conflict of Interest Statement

The authors declare that the research was conducted in the absence of any commercial or financial relationships that could be construed as a potential conflict of interest.
